# Advancing institutionalization and sustainability of field epidemiology training programs in the Eastern Mediterranean region

**DOI:** 10.3389/fpubh.2025.1669319

**Published:** 2025-10-01

**Authors:** Yousef Khader, Ali Ahmed Al-waleedi, Sheikh Abdulhafed Al-Shoteri, Adil Said Al Wahaibi, Ahmad Farshid Muhammadi, Haitham Bashier, Ayman Bani Mousa, Ramez Dwekat, Shamaila Usman, Abdulla Bin-Ghouth, Hassan Chrifi, Tareq Al-Hawwash, Hajer Letaief, Zainab Naseer Abbas, Ruba Kamal Alsouri, Mohammed Akrim, Mohannad Al Nsour

**Affiliations:** ^1^Department of Public Health, Jordan University of Science and Technology, Irbid, Jordan; ^2^Department of Community Medicine and Public Health, Aden University, Aden, Yemen; ^3^Faculty of Medicine and Health Sciences, University of Aden, Aden, Yemen; ^4^Department of Surveillance, Ministry of Health, Muscat, Oman; ^5^Public Health and Management Training Department, Afghanistan National Public Health Institute, Ministry of Public Health, Kabul, Afghanistan; ^6^Public Health Emergency Management Center, The Eastern Mediterranean Public Health Network, Amman, Jordan; ^7^Epidemic Administration Directorate, Jordan Ministry of Health, Amman, Jordan; ^8^Nablus Health Directorate Health Directorates, Palestine Ministry of Health, Ramalla, Palestine; ^9^CDC Department, National Health Institute, Kabul, Pakistan; ^10^Yemen National Public Health Institute, Aden, Yemen; ^11^National School of Public Health, Ministry of Health and Social Protection, Rabat, Morocco; ^12^Primary Health Care Departments, Palestine Ministry of Health, Ramalla, Palestine; ^13^National Observatory of New and Emerging Diseases, Tunis, Tunisia; ^14^Iraq FETP Program, Iraq Ministry of Health, Baghdad, Iraq

**Keywords:** field epidemiology training program, institutionalization, sustainability, funding models, career pathways, accreditation, EMPHNET

## Abstract

**Introduction:**

Sustaining Field Epidemiology Training Programs (FETPs) is critical for long-term public health capacity. Institutionalization—embedding programs within national health systems—is a major step toward sustainability. This manuscript explores the experiences, perceived challenges, and strategies related to the institutionalization, sustainability, and funding of FETPs in the Eastern Mediterranean Region (EMR) and offers recommendations to strengthen their long-term integration within national health systems.

**Methods:**

A participatory regional workshop was held in Amman from May 18–20, 2025, to review frameworks, share country experiences, and develop sustainability plans. Participants included FETP directors, ministry officials, and alumni from nine countries. Sessions addressed governance, financing, accreditation, career pathways, and stakeholder engagement. Data were synthesized thematically from session notes and program documents.

**Results:**

Twenty-eight participants representing Afghanistan, Iraq, Jordan, Pakistan, Palestine, Tunisia, Morocco, Oman, and Yemen attended the regional workshop. Most were experienced public health professionals and FETP graduates. Country teams highlighted the importance and impact of FETP, while funding constraints and undefined career tracks were common challenges. Institutionalization, defined as embedding FETPs into national strategies with government ownership, legal frameworks, and dedicated financing, emerged as a critical priority. Participants recommended shifting to mixed financing models, pursuing accreditation, and linking programs to universities. The lack of career pathways underscore the need for policies recognizing FETP qualifications in promotions. Stakeholder engagement and advocacy were identified as essential for sustaining support.

**Conclusion:**

Sustaining FETPs requires deliberate country-led action, stable funding, accreditation, and clear career progression. With committed leadership and regional collaboration, FETPs can evolve into permanent pillars of public health capacity and health security.

## Introduction

Field Epidemiology Training Programs (FETPs) were adapted from the U.S. Centers for Disease Control and Prevention (CDC) Epidemiologic Intelligence Service (EIS) in 1980 to rapidly build epidemiologic capacity globally (1). These competency-based, “learning-by-doing” programs train health professionals in applied epidemiology through field assignments supplemented by classroom learning. Approximately 75% of the training period is dedicated to fieldwork under direct supervision, while the remaining 25% consists of formal didactic sessions (2). Graduates have measurably strengthened public health surveillance, outbreak response, and data systems (3–8). Their roles during COVID-19 underscore their value in health emergency management (8, 9). In the Eastern Mediterranean Region (EMR), FETPs operate at all three tiers (Advanced 2-year, Intermediate 1-year, Frontline 3-month) to build a skilled epidemiology workforce tailored to each country’s context. Through the Eastern Mediterranean Public Health Network (EMPHNET), the US CDC and partners support FETPs in 17 EMR countries. Collectively, EMR FETPs have produced thousands of graduates who have contributed to regional health security by improving disease surveillance and public health decision-making during crises.

Despite these achievements, FETPs in the EMR continue to face significant challenges in both institutionalization and sustainability. Institutionalization refers to the formal integration of FETPs into national health systems through legal recognition, administrative structures, and dedicated financial mechanisms. Sustainability denotes the long-term capacity of these programs to function effectively and adapt to evolving public health needs, supported by stable financing, quality assurance, and defined career pathways. Institutionalization is, therefore, a foundational step toward achieving sustainability. However, many programs remain heavily donor-dependent, lack formal integration within ministries of health or national public health institutes (NPHIs), and are constrained by unclear career trajectories and inadequate domestic budget allocations.

In the EMR, most FETPs remain financed predominantly by external donors, particularly in fragile or conflict-affected contexts such as Afghanistan and Yemen. Only a handful of countries—including Jordan, Iraq, Morocco, and Saudi Arabia—have institutionalized domestic funding through their ministries of health. Mixed financing approaches, such as university partnerships and service contracts, are emerging but are not yet widespread. With donor support expected to decline, prioritizing dedicated government budget lines has become a regional imperative for sustaining FETPs.

Previous literature emphasizes that institutionalization, political commitment, and funding are primary factors for FETP sustainability (10). The Global Field Epidemiology Roadmap also calls for formal career paths and workforce targets to ensure retention of skilled epidemiologists (11). This manuscript aims to explore and document the experiences, challenges, and strategies related to the institutionalization, sustainability and funding of FETPs in the EMR, and to develop actionable recommendations to strengthen their long-term effectiveness and integration within national public health systems.

## Methods

A participatory workshop design was employed. EMPHNET convened a regional workshop on FETP sustainability, institutionalization, and funding in the EMR from May 18–20, 2025, in Amman. The objectives were to review conceptual frameworks and best practices for sustaining FETPs, share country experiences, including gaps and progress, and develop actionable sustainability plans.

Participants included FETP directors, ministry officials, and alumni from nine countries (Afghanistan, Iraq, Jordan, Pakistan, Palestine, Tunisia, Morocco, Oman, and Yemen). EMPHNET staff and invited experts introduced five core dimensions of sustainability: (1) governance and institutionalization, (2) funding and financing, (3) quality assurance and accreditation, (4) career pathways and professional development, and (5) stakeholder engagement and advocacy.

On day 1, a plenary lecture outlined these dimensions, emphasizing the importance of “country-owned, country-led” programs. Country teams (three to five delegates each) presented status reports. Over the following 2 days, sessions addressed institutionalization (definitions, case examples, and panel discussions), quality and accreditation, funding models, stakeholder mapping, career pathways, visibility and advocacy, and strategy development.

Detailed notes from plenary and group discussions were documented by designated rapporteurs and consolidated into session summaries. Data were synthesized thematically by two reviewers, who independently identified and grouped recurring themes, with discrepancies resolved through discussion. Participants from all nine countries endorsed the identified themes, demonstrating full consensus across the region. Findings represent the perspectives of workshop participants and should be interpreted as reflecting their experiences rather than generalizable population data No formal human subjects’ research was conducted, as this was a professional workshop; the analysis is based on program documents and participant discussions. Direct quotations were not used; rather, consensus findings and illustrative examples were reported. Findings were triangulated with existing FETP literature and EMPHNET reports to ensure consistency.

## Results

### Participants’ characteristics

Twenty-eight participants attended the workshop, representing nine EMR countries (Afghanistan, Iraq, Jordan, Pakistan, Palestine, Tunisia, Morocco, Oman, and Yemen). All participants held at least a bachelor’s degree. Their professional roles included FETP directors (n = 7), FETP coordinators (n = 6), technical advisors (n = 4), and senior officials from ministries of health—such as surveillance heads and deputy ministers—as well as public health institutions (n = 11). Many were also graduates of the FETP. Their areas of expertise spanned epidemiology, community/public health, infectious/tropical medicine, and preventive medicine.

### Country experience: highlights

All country teams noted the value of “learning by doing” in emergency responses. Several presentations highlighted FETP roles in recent outbreaks (e.g., COVID-19, cholera) even if data were not formally compiled. Delegates agreed that joint regional initiatives (shared courses, mentorship exchanges) could fill gaps in small programs. During country presentations and discussions, delegations reported progress and challenges in their contexts. Funding was uniformly reported as a relative weakness. All participants identified insufficiently defined career tracks for graduates and mentors, reported that accreditation and formal evaluation were often incomplete, and mentioned that advocacy efforts were generally limited at the country level. Table 1 shows the comparative status of FETP institutionalization, financing, accreditation, and career pathways across nine countries in the Eastern Mediterranean Region.

**Table 1:** Comparative status of FETP institutionalization, financing, accreditation, and career pathways across nine countries in the Eastern Mediterranean Region.

Country	Institutionalization status	Financing (current/typical)	Accreditation (program-level)	Career pathways
Afghanistan	Active in MoPH but remains donor-dependent; lacks policy, financing, and legislation for institutionalization.	Predominantly donor-funded.	In progress	Limited; lack of defined tracks
Iraq	Embedded in the MoH Department of Epidemiology; partnered with Baghdad University	Domestic funding institutionalized	Accredited	Defined/strong—civil-service roles support progression.
Jordan	Fully institutionalized—integrated into the Community Medicine specialty; many MoH directorates led by alumni.	Domestic funding institutionalized	Not Accredited	Strong—integration enhanced career prospects/demand.
Pakistan	Partial—decentralized system and fragmented ownership hinder funding.	Mixed/donor-reliant	Accredited (2-year program)	Limited; undefined tracks
Palestine	In planning phase	Not applicable	Not applicable	Not applicable
Tunisia	Housed in MoH; aligned with workforce plans; active alumni network.	Lack of sustainable government financing.	Not Accredited	Lacks formally defined career tracks for graduates
Morocco	Fully institutionalized—government-backed; curriculum expanding.	Domestic funding institutionalized	Accredited (2- year program)	Career progression pathways not clearly documented
Oman	No clear structure for the program and no dedicated staff.	Donor funded	Not applicable, as only a frontline FETP has been implemented to date	Not clearly defined yet
Yemen	FETP is institutionalized within the Yemen National Public Health Institute. Constrained by conflict; reliant on external aid.	Predominantly donor-funded.	Not Accredited	Undefined tracks noted across countries.

### Institutionalization within National Health Systems

A central theme of the workshop was the institutionalization of FETPs, which participants defined as the permanent embedding of these programs within national health system structures. Key principles emphasized included government ownership, reflected through leadership by ministries of health (MoH) or NPHIs; legal recognition, through policies or legislation explicitly citing FETPs; and sustainable financing secured through government budgets. Participants noted that strong institutionalization requires coordinated action across several domains: (1) Policy integration—incorporating FETP training into national health workforce development plans and civil service regulations, including recognition of FETP in promotion criteria; (2) Dedicated budget lines—establishing permanent FETP budget allocations within MoH structures to ensure continuity regardless of donor support; and (3) Academic linkages—accrediting FETP curricula through universities or offering joint certification to enhance the programs’ legitimacy and sustainability.

A panel discussion reviewed progress in institutionalizing FETPs across EMR countries, highlighting varied levels shaped by national contexts. Jordan’s FETP, among the oldest (1998), is fully institutionalized, integrated into the national Community Medicine specialty. Residents complete 2 years in the advanced FETP followed by 2 years of clinical rotations, qualifying as community medicine specialists. Nearly all MoH directorates are now led by FETP alumni, strengthening governance and elevating the program’s profile. This integration has enhanced career prospects and demand. Iraq has similarly achieved high institutionalization. Established in 2010, its FETP is embedded in the MoH’s Department of Epidemiology and partnered with Baghdad University from inception. Defined civil service roles have anchored alumni in the health system. Morocco’s FETP is also fully institutionalized, backed by government support, and expanding its curriculum with new modules. Saudi Arabia’s FETP is well institutionalized, recently shifting governance from the MoH to the Public Health Authority.

Tunisia has demonstrated moderate progress in institutionalizing its FETP. The program is housed within the Ministry of Health and aligned with national workforce development plans, with additional support provided through continuing education activities and an active alumni network. However, challenges remain, particularly the absence of sustainable government financing and formal academic accreditation. Pakistan’s FETP remains partly institutionalized due to a decentralized health system and fragmented ownership, which hinders funding. Despite this, it continues to graduate epidemiologists and support surveillance. Afghanistan’s FETP, active in the Ministry of Public Health, has trained many epidemiologists but remains donor-dependent, lacking policy, financing, and legislation to ensure institutionalization.

Oman’s FETP, launched in 2023, has successfully completed four Frontline FETP cohorts, with the majority of graduates subsequently integrated into the national health system. Early challenges include formalizing governance, securing funding, and building partnerships. Yemen’s FETP (2011) has been constrained by conflict, leaving it weakly institutionalized and reliant on external aid, though it continues to adapt training and seek visibility. Palestine is in the earliest stage, having completed a Training Needs Assessment and planning to launch its first Frontline FETP cohorts by late 2025.

### Funding models and challenges

Workshop participants uniformly identified financing as a core sustainability dimension. Few countries have dedicated MoH budgets for FETP. In the discussion, participants recommended a shift away from donor dependence towards mixed financing models. Key points included (1) Domestic Budgeting: Governments should allocate specific line items for FETP training and operations. This may require demonstrating FETP value to finance ministries. Delegates agreed that embedding FETP costs in national health budgets (or in donor transition plans) is essential for stability; (2) Service Provision: FETPs were encouraged to “position themselves as service providers” by offering epidemiological consultancy, outbreak investigation services, or training courses to generate revenue; (3) Partnerships: Engaging private sector and academic partners can diversify funding. Suggestions included joint degree programs (residents pay tuition as part of an MPH) and public-private training courses. Cross-border grants and regional proposal writing were also noted; (4) Efficiencies: Participants proposed cost-sharing mechanisms, such as training of trainers (ToT) to build local faculty and reduce dependence on expensive international instructors. They cautioned that support must be balanced across FETP tiers (advanced, intermediate, frontline) to avoid weakening the broader surveillance system.

Recognizing a decline in funding, participants stressed the urgency of alternative revenue. One outcome was the idea of establishing a regional sustainability fund, potentially managed by EMPHNET, to provide seed grants for country plans (analogous to “pooled donor funds”). Despite creative models, delegates agreed that domestic financing must eventually dominate. Political commitment (e.g., ministerial endorsements of FETP funding) was seen as pivotal.

### Quality assurance and accreditation

Quality assurance mechanisms are vital for program credibility. The workshop participants advocated for FETPs to adopt international standards. The TEPHINET accreditation process was highlighted as a tool to align programs with best practices. Participants noted that accreditation reviews prompt strengthening mentorship tracking, supervision, and documentation. Accreditation also serves as an advocacy function: it provides “evidence of the program’s value” to stakeholders. Delegates agreed that achieving accreditation should be pursued, even if it requires technical support (e.g., employing accreditation consultants) to meet criteria. Additionally, linking FETPs with universities to award formal diplomas or degrees (joint certification) was discussed as a way to enhance legitimacy.

Beyond accreditation, routine monitoring & evaluation were emphasized. Countries were encouraged to implement periodic program evaluations (e.g., by external reviewers or embedded monitoring and evaluation units) and integrate FETP indicators into national health metrics. This creates accountability for maintaining training quality.

### Career pathways and professional development

A recurrent theme was the lack of formal career pathways for FETP alumni and mentors. The workshop introduced the idea that successful training programs should enhance graduates’ career prospects. Many countries reported that FETP was treated as a short-term training rather than a career pipeline. For instance, Jordan requires graduates to already be MoH employees, limiting entry diversity. Participants noted that without clear job ladders, many graduates either stagnate or leave the field. Session discussions on “FETP Career Pathway” highlighted the need for national policies recognizing FETP qualifications as professional credentials. Because current evidence on alumni careers is scarce, participants reported that ministries should revise civil service promotions to reward FETP certification, and human resources departments should reserve epidemiology positions for FETP alumni.

Professional development was also addressed. Continuous mentorship networks were proposed, with senior graduates mentoring new cohorts across countries. Regional ToT programs (e.g., in scientific writing, leadership) were recommended to keep alumni engaged and competent. Participants stressed that investing in career development (e.g., funding fellowships, conference travel) would increase retention. Therefore, establishing a clear epidemiology career track—with corresponding titles and salary scales—was seen as essential for long-term program sustainability.

### Stakeholder engagement and advocacy

The workshop underscored that FETP sustainability is as political as technical. High-level advocacy and visibility were identified as cross-cutting factors that can unlock resources and commitment. The final session focused on “Increasing FETP Visibility and Advocacy” and outlined strategic steps for promoting programs. Participants defined visibility as broad awareness of FETP accomplishments (social media presence, press coverage, conference abstracts) and advocacy as actions to influence decision-makers. Key messages included that greater visibility leads to funding and institutional support. EMPHNET’s existing efforts—such as quarterly newsletters, webinars, and a biennial conference—were praised. Workshop delegates suggested further actions: publishing annual country impact reports, creating success-story policy briefs, engaging alumni as “FETP ambassadors,” and using social media campaigns.

In open discussion, countries noted examples of advocacy: one FETP leader secured local media coverage during an outbreak investigation, which raised public interest. Participants agreed to coordinate regional messaging: for instance, a joint statement on FETP contributions to COVID-19. Critically, it was emphasized that engaging stakeholders early (MoH leadership, academia, donors) through steering committees ensures continued buy. Workshop participants concluded that “increasing FETP visibility and advocating for country programs” directly increases the chances of partnerships and funding.

## Discussion

The workshop findings reaffirm that FETPs are recognized as assets. Graduates continue to respond to multiple emergencies and have improved health policies in their countries. FETP alumni occupy leadership roles in ministries and contribute to outbreak control. However, sustaining these programs requires moving beyond crisis-driven support to systematic integration. The findings of this workshop echo other regions’ experiences (10). A recent analysis found that FETPs require embedding in government systems for long-term success (3). Our workshop highlighted specific steps that operationalize embedding.

As institutionalization is an ongoing effort, countries and regional partners have recognized the need for structured follow-up. Plans are underway to work with ministries of health and academic institutions to develop formal institutionalization and sustainability plans, which will include financing mechanisms, accreditation processes, and workforce integration strategies. These plans are expected to capture lessons learned and identify best practices, allowing countries at different stages of institutionalization to benefit from shared experiences. The regional commitment to generating and disseminating such knowledge will not only inform country-level strategies but will also contribute to building a stronger collective framework for sustaining FETPs in the region.

The need for clear career pathways is repeatedly cited in FETP guidance and literature (10, 11). FETP graduates worldwide are engaged in ministries, non-governmental organizations, and academia, but tracking their progression is challenging. Without defined career progression, there is a risk that trained epidemiologists will leave their positions or have their expertise misapplied. Establishing national competency frameworks could help ensure that FETP training translates into recognized job functions. Retention of FETP alumni emerged as a critical factor in advancing institutionalization across the region. Where clear career trajectories exist, such as in Jordan, Iraq, and Morocco, alumni have moved into leadership positions within ministries of health, provincial health directorates, and national surveillance programs. These pathways not only enhance individual career prospects but also strengthen the institutional capacity of health systems. In contrast, countries where government structures do not provide permanent positions for graduates face significant challenges in retaining alumni. The absence of defined career ladders or incentives often results in the loss of skilled professionals to other sectors or international organizations.

Diversifying financial sources was a consensus priority. Consistent with previous recommendations (12), workshop delegates emphasized government budget lines, supplemented by innovative revenue (consulting, courses) (10). The suggestion to treat FETP as a service provider is notable and parallels models in Africa where programs offer paid surveillance training or epidemic support. The balanced-tier approach (not neglecting frontline/intermediate levels) aligns with ensuring broad health system resilience. Experience from other regions (13) confirms that local financing and multi-sector partnerships are key to sustainability.

The focus on TEPHINET accreditation and joint academic degrees reflects a global movement towards standardization. Accreditation not only raises quality but provides an advocacy tool by signaling that a program meets international benchmarks. This workshop reiterated that embedding quality assurance into national health performance metrics can legitimize FETP as a core competency-building program.

Perhaps most unique in this workshop was the structured attention to visibility and advocacy. While regional networks like EMPHNET routinely communicate FETP successes, participants received concrete tools (e.g., messaging plans, media outreach) to do so locally. This emphasis is justified: our findings align with FETP advocates’ calls (14) to market FETPs for political support. High visibility was explicitly linked to increased funding opportunities; a concept echoed in CDC’s framework for sustainability, which notes the importance of champions and awareness.

Our analysis draws on both the workshop and the published literature to converge on similar themes: integration into health systems, stable financing, professional development, and quality. These dimensions are interconnected. For example, institutionalization (governance) enables funding, which supports human resources development and quality assurance. The workshop’s multi-dimensional approach reflects this interplay. Political stability, economic capacity, and health system organization all modulate how easily FETPs can be sustained.

Globally, FETPs vary in their level of institutionalization. Programs with strong government ownership, legal frameworks, domestic financing, and integration into national workforce and academic systems are generally more sustainable and credible, while those reliant on donor funding and lacking policy support or national integration remain less institutionalized and more vulnerable to change. Experiences from other regions highlight important contrasts and lessons. In Africa, FETPs are increasingly embedded within government public health institutions; for example, the Zambia FETP was established within the Ministry of Health’s Zambia National Public Health Institute (15), and Ghana’s FELTP is run in partnership with the University of Ghana (16). Such academic integration and national ownership have helped institutionalize these programs, with graduates deliberately placed as epidemiologists in disease control programs at national and subnational levels. Regionally, AFENET has been instrumental in fostering country ownership and program quality, although establishing formal career tracks for graduates remains a challenge. In Latin America, Brazil’s program is a prime example of deep institutionalization (17), being fully managed by the Ministry and supported through a local funding mechanism to cover trainee stipends. Similar patterns exist elsewhere in the region, where FETPs are housed within national institutes of health or universities and often confer academic degrees or certificates. In the Eastern Mediterranean Region (EMR), FETPs are striving for similar goals, with several programs pursuing accreditation to strengthen quality and sustainability. Nonetheless, financing and career pathways remain pressing challenges: many EMR FETPs began with donor funding, and ministries must now absorb costs to sustain training cohorts and staff. Some countries have started allocating government funds or creating dedicated budget lines, echoing institutionalization efforts in Zambia and Costa Rica. However, career pathways for graduates require further development, as global experience shows that without clear posts and advancement opportunities, FETP graduates risk being underutilized or leaving the system (18). EMR health authorities are working to address this by exploring official recognition of FETP graduates as specialists or by creating epidemiologist positions at national and subnational levels. Overall, global experience indicates that despite contextual differences, the foundations of FETP sustainability are consistent across regions: strong political commitment, integration into public health institutions, domestic financing, academic or professional accreditation, and structured career pathways that maximize the use of graduates’ skills.

This study has several limitations. The analysis is based on participant discussions and documents from a single regional workshop, and therefore reflects participant perspectives rather than systematically collected data. While participants represented a range of countries, the findings may not capture the full diversity of experiences across the region. Nonetheless, the themes align with prior evaluations and provide valuable insights into institutionalization and sustainability challenges. While the workshop included directors and technical staff, ultimate authority often rests with ministers and senior budget officials. This underscores the importance of engaging such decision-makers in future workshops and advocacy efforts to secure the political buy-in needed for sustainable financing.

In conclusion, workshop findings and literature together make clear that FETP sustainability cannot be assumed. These programs, though impactful, require deliberate policy action to become permanent assets. The EMR, like other regions, must therefore invest strategically in embedding FETPs—by codifying their role, securing budgets, supporting career growth, and branding their contributions—to reap long-term health security dividends. Based on workshop outcomes and global best practices, the following policy and practice recommendations for EMR governments, FETP programs, and partners are proposed:

- Integrate FETP into national health and human resources plans. For example, amend public health laws/regulations to formally recognize FETP training and graduates’ roles in disease control. Establish an intersectoral steering committee (MoH, academia, donors) to oversee FETP strategy and funding allocations. Ensure inclusion of FETP in emergency preparedness plans and regular MoH operations.
- With international funding declining, domestic resources should be prioritized for core FETP activities. External funds, if available, may support special projects. Governments are encouraged to establish dedicated FETP budget lines (e.g., within the MoH or NPHI) to cover training costs and graduate deployment.
- Define a clear career progression for FETP graduates within the civil service. Ensure that completing FETP (at any tier) confers priority in promotion or placement for surveillance and outbreak positions. Work with HR departments to revise job descriptions and promotion criteria to explicitly include FETP qualifications. Foster in-service mentorship by pairing graduates with senior epidemiologists to support ongoing professional development. Consider creating fellowship opportunities to retain high performers.
- Pursue formal accreditation (TEPHINET or regional equivalent) for each FETP. Build internal capacity (data systems, mentoring documentation, curricula review) to meet accreditation standards. Implement routine evaluations: annual performance reviews and periodic external assessments. Link evaluation outcomes to continuous improvement: update curricula regularly based on evaluation findings and emerging health threats.
- Partner with local universities to offer joint academic credentials (e.g., MPH degrees with FETP practicum). This enhances the program’s credibility and may open tuition-based funding (universities contributing faculty or resources). Integrate FETP modules into university curricula to reinforce the training.
- Train FETP directors and staff on policy issues, with a focus on effective communication with non-scientific stakeholders such as ministries of finance and human resources.
- Develop and implement advocacy and communications plans at national and regional levels. This plan should use media (social, traditional), success stories, and events (e.g., field epidemiology day) to raise FETP profiles. Engage parliamentarians, health influencers, and community leaders by organizing briefings on FETP’s role in public health. Establish an FETP alumni association to serve as ambassadors. Leverage EMPHNET’s regional platforms (newsletter, conferences) to showcase country achievements and attract support.
- FETPs should implement a system to track alumni, as they represent a valuable resource for responders, mentors, and faculty.
- Invest in faculty and mentorship development. Provide opportunities for FETP mentors to attend training-of-trainers (ToT) workshops to refresh their skills. Use regional networks for peer exchanges and joint training sessions. Encourage multi-country project collaborations to keep graduates engaged and demonstrate value.
- Tailor FETP tiers for evolving needs (e.g., outbreak detection, Non-Communicable Diseases, One Health). Expand new modalities by forming inter-country cohorts. Ensure curricula remain updated to address regional health priorities.
- Establish key indicators for FETP sustainability. Monitor progress annually and adjust strategies. Share this data in regional meetings to promote accountability.

Implementation of these recommendations should be country-driven but supported regionally. EMPHNET and partners can facilitate by providing technical assistance (e.g., drafting policy briefs, conducting workshops on advocacy, offering accreditation guidance). EMPHNET will also maintain close follow-up with participating countries to support the development of institutionalization and sustainability plans, while capturing and disseminating lessons learned and best practices. A collaborative regional framework can harmonize standards while allowing for national customization. In essence, governments and stakeholders must commit resources and leadership to transform FETPs from donor-dependent projects into permanent pillars of the public health workforce.

## Data Availability

The data analyzed in this study is subject to the following licenses/restrictions: data supporting the findings of this study are available from the corresponding author upon request. Requests to access these datasets should be directed to YK, yskhader@just.edu.jo.
